# The Use of Complementary and Alternative Medicine among Peritoneal Dialysis Patients at a Second-Level Hospital in Yucatán Mexico

**DOI:** 10.3390/healthcare11050722

**Published:** 2023-03-01

**Authors:** Carlos Gracida-Osorno, Sandra Luz Jiménez-Martínez, Andrés Humberto Uc-Cachón, Gloria María Molina-Salinas

**Affiliations:** 1 Servicio de Nefrología, Hospital General Regional No. 1, Instituto Mexicano del Seguro Social, Mérida 97150, Yucatán, Mexico; 2 Unidad de Investigación Médica Yucatán, Unidad Médica de Alta Especialidad, Instituto Mexicano del Seguro Social, Mérida 97150, Yucatán, Mexico

**Keywords:** chronic kidney disease, peritoneal dialysis, complementary medicine, alternative medicine

## Abstract

Background: Complementary and alternative medicine (CAM) is widely used for multiple reasons such as treatment of diseases and their symptoms, empowerment, self-care, disease prevention, dissatisfaction, adverse effects or cost of conventional medicine, perception of compatibility with beliefs, and idiosyncrasy. This study investigated CAM use in patients with chronic kidney disease (CKD) undergoing peritoneal dialysis (PD). Methods: A cross-sectional survey study was conducted with 240 eligible patients with CKD in the PD program. By applying the I-CAM-Q-questionnaire, the frequency, level of satisfaction, and reasons for CAM use were explored, and the demographic and clinical data of users and non-users were analyzed. Data analysis included descriptive analysis, Student’s *t*-test, Mann-Whitney U, chi-square, and Fisher tests. Results: The main types of CAM used were herbal medicine, and chamomile was the most commonly used. To improve the state of well-being was the main reason for use, the attributable benefit of CAM was high, and only a low percentage of users reported side effects. Only 31.8% of the users informed their physicians. Conclusion: The use of CAM is popular among renal patients, and physicians are not adequately informed; in particular, the CAM type ingested represents a risk for drug interactions and toxicity.

## 1. Introduction

Chronic kidney disease (CKD) is a condition that leads to disability, decreased quality of life, and substantial social and financial costs. It is now recognized as a global public health priority that has reached epidemic proportions worldwide, with a consequent impact on morbidity and mortality and cost to the health system. In 2017, the global prevalence of CKD was 9.1%, and in 2019, the Pan American Health Organization estimated that it is a leading cause of disease burden, categorized as the 8th cause of mortality and the 10th cause of years of life lost in both sexes [1,2].

CKD is more prevalent in people with obesity, hypertension, and/or diabetes mellitus as well as elderly people, women, and racial minorities and is expected to increase, including the stage with the requirement of a renal replacement therapy, namely peritoneal dialysis (PD) [3,4].

Approximately 80% of the world’s population uses complementary and alternative medicine (CAM) to maintain their health [5,6]. The use of CAM by the population has experienced significant growth in the last 15 years, with consequent medical, economic, and sociological impacts; this increase is especially evident in individuals with chronic diseases. Patients mention using it for multiple reasons, such as for the treatment of diseases and their symptoms, but also for the maintenance of health, empowerment, self-care, disease prevention, improvement of quality-of-life dissatisfaction with allopathic medicine, adverse effects of the medications, and their cost. Additional reasons include the need for the patient to control the evolution of their disease and the perception of compatibility of the use of CAM with their values, beliefs, and idiosyncrasy [7,8,9,10].

Studies conducted to date suggest that adult patients with chronic diseases such as diabetes mellitus, systemic arterial hypertension, chronic kidney disease, cancer, chronic obstructive pulmonary disease, and rheumatoid arthritis, among others, are more prone to the use of CAM as part of self-care and management of their condition and are more likely to use CAM at greater comorbidity [11,12,13,14,15].

Doctors are often inadequately informed by their patients about their CAM use; for example, only 18% of Polish cancer patients discussed using CAM with a doctor [16] in contrast with the 60% of pediatric oncology patients from Switzerland that discussed use of CAM with their oncologist [17] and the 31.3% of Colombian rheumatic patients who use CAM and informed their rheumatologists because of fear of retaliation [18].

In Mexico, only a few studies have been carried out that described the use of CAM in this population. Carmona-Sanchez reported use of CAM to treat various digestive disorders (prevalence 28–51%) [19]; Herrera-Arellano et al. reported that 73.4% of HIV-positive patients were users of some type of CAM [20], and the cactus—nopal—was the most common indigenous remedy used to treat diabetes mellitus (73.1%). The geographic region of the Yucatán Peninsula was the region of founding of the Mayan culture, which reached an important degree of development in the field of traditional medicine, characterized using various preparations of plants, animals, and minerals with curative action by traditional doctors or healers; in addition, the exercise of Mayan medicine was entrusted to three specialists of different ranks: the h-men (priest), dza-dzac (the one who heals with herbs), and the pulyah (sorcerer) [21]. Mayan medicine continues to be used both in rural and urban-zone populations of the Yucatán Peninsula.

The use of CAM in patients with chronic diseases has become an interesting topic for academics and for medical doctors. Patients with chronic diseases generally have more than one type of ailment, so it is very important that physicians know and understand the reasons that influence patients to use any type of CAM, with the aim to be their guide in decisions to better treatments and consequently aid health care systems. The absence of adequate information from patients to physicians may also be related to social perceptions [22,23].

CAM is widely used in the general population, and its use in patients with CKD has been only slightly investigated worldwide. Patients with PD may be more likely to be users of CAM in view of the chronicity of their disease and the various comorbidities. Moreover, this group of patients could be at an increased risk of drug interactions, toxicity, or electrolyte disturbances owing to the absence of renal excretory function.

The prevalence and types of CAM used among Mexican CKD patients are unknown. Therefore, the present survey was designed to document the frequency and types of CAM usage in patients with CKD treated with PD who attended at a regional hospital in Yucatán, Mexico, using a validated questionnaire. This study also investigated the report to their doctors about the use of CAM, reason for use, and perception of the benefit of patients.

## 2. Materials and Methods

### 2.1. Study Design and Setting

This is a cross-sectional study. Data collection was conducted among patients with CKD who received PD and were invited to participate for a phone call, and in the medical appointment follow-up, the patients were interviewed face-to-face by two experienced researchers. The study was conducted at the Dialysis Clinic in the Regional General Hospital “Ignacio García Téllez” of the Instituto Mexicano del Seguro Social (IMSS), a second-level hospital located in Mérida Yucatán, Mexico, from November to December 2018.

### 2.2. Study Population

A total of 319 patients from the PD program of the Ignacio García Téllez Hospital were invited to participate in this study (*n* = 319). The inclusion criteria were an age older than 18 years, with at least one clinical evaluation by a nephrologist of the PD program within the last two months, and who agreed to participate with verbal answers to the questionnaire by interview. The exclusion criteria were as follows: renal transplant, age < 18 years old, refusal to participate, undergoing hemodialysis, deceased during the study period, and did not attend the appointment.

### 2.3. Sample Size

**Figure 1:**
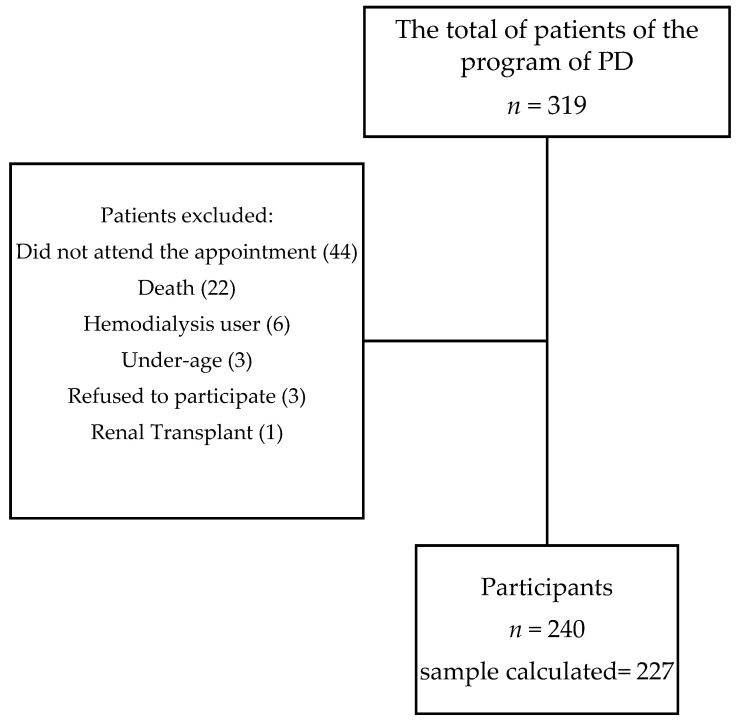
The study population selection. PD, peritoneal dialysis.

The sample size for survey was estimated using an 18% anticipated prevalence of use of CAM for dialysis patients [24], and with a 95% level of confidence and a 5% margin of error, the estimated sample size was 227 (Epi Info v. 7.2 CDC, Atlanta, GA, USA). The final sample was comprised of 240 PD patients from our hospital (Figure 1).

### 2.4. Research Instrument

The data were collected by a questionnaire previously reported and validated and known as I-CAM-Q (the International Complementary and Alternative Medicine Questionnaire), which includes four parts: (a) examination by health provider, (b) complementary treatments, (c) use of herbal medicine and dietary supplements, and (d) self-help practices [25,26] (Supplementary Materials).

### 2.5. Clinical Characteristics and Biochemical Parameters of Patients

The clinical characteristics of patients were obtained during the medical appointment by clinical exploration.

The biochemical parameters of the patients were obtained from their clinical appointment and corresponded to previous bimonthly medical appointments. After at least 12 h fasting, venous blood samples were collected to measure the complete blood count and various biochemical components (glucose, creatinine, urea, uric acid, albumin, calcium, phosphorus, and potassium).

### 2.6. Ethics

The project was approved by the local committee of Investigation and Ethic 3201 of Regional General Hospital Ignacio García Tellez IMSS (registered number R-2018-3201-26). The participants were given information about the aim of the study and the content of the questionnaire. Informed consent was obtained from all patients before confirming their participation in this investigation.

### 2.7. Data Analysis

Continuous variables are expressed as arithmetic mean ±1 standard deviation (±1SD), and categorical variables are presented as frequencies and percentages. For comparison and analysis, the study population was divided into two groups: CAM users and non-CAM users. Continuous variables were analyzed using the Student’s *t*-test or Mann-Whitney’s U test depending on the normality distribution of the data, while the categorical variables of the two groups were analyzed using the chi-square test or Fisher’s exact test. Differences were considered statistically significant at *p*-value < 0.05.

## 3. Results

### 3.1. Use of CAM

The sociodemographic characteristics of patients are shown in Table 1. The frequency of CAM use in the study population was 55.0% (132/240), of which 50.8% (67/132) were male and 49.2% (65/132) women. No statistically significant differences were observed between the sociodemographic characteristics of the CAM users and non-users (Table 1).

### 3.2. Type of CAM

The most common type of CAM used by patients was herbal medicine (50.0%, 66/132), followed by mind-body practices such as music therapy (24.2%, 32/132) and relaxation techniques (18.9%, 25/132) (Table 2).

**Figure 2:**
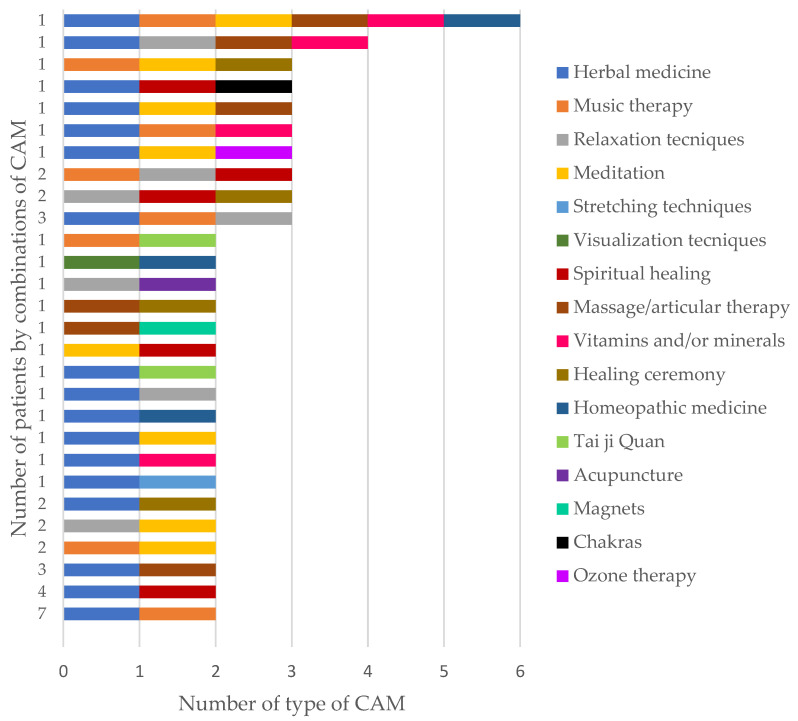
Combinations of type of complementary and alternative medicine (CAM) employed by the patients.

Most of our study population (65.1%; 86/132) referred to using only one type of CAM, while 34.8% (46/132) of patients used more than one CAM in combination; the majority of them used two types of CAM (67.4%; 31/46), followed by three (28.3%; 13/46). The most frequent combination of CAM was herbal medicine and music therapy, followed by herbal medicine and spiritual healing. The distribution of the combinations of CAM types used by the study population is shown in Figure 2.

Regarding herbal medicine, the patients used more than two types of herbal products (40.9%; 27/66), such as teas, referencing a total 36 different types of herbs and natural products. The most frequently used was chamomile with 25.8% (25/97), followed by moringa leaves with 16.0% (15/97), chaya leaves with 8.5% (8/97), and orange leaves with 6.2% (6/97) (Table 3).

### 3.3. Reason to Use CAM

**Figure 3:**
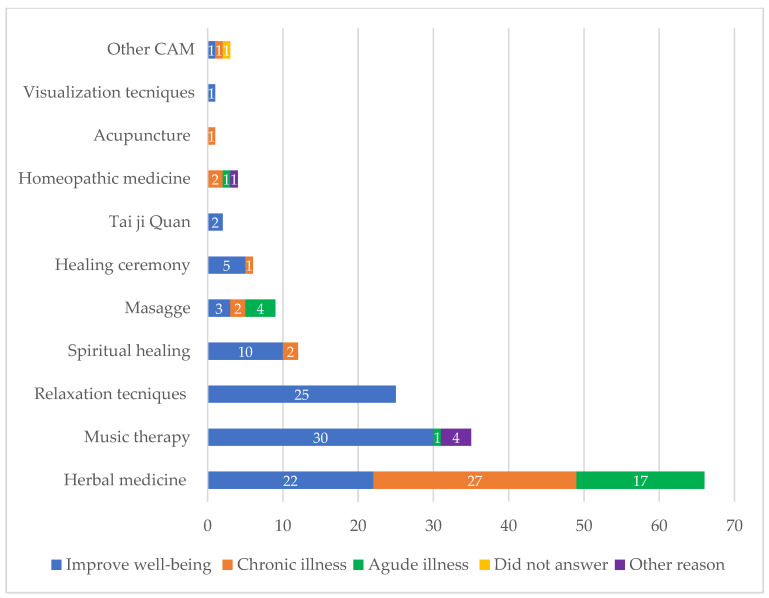
Reasons reported by patients for using complementary and alternative medicine (CAM).

The most frequent reason for using CAM was to improve well-being (71.7%; 132/184). This reason was referred to by 100% of practitioners of relaxation techniques (25/25), meditation (9/9), and Tai Ji Quan (2/2); 85.7% of practitioners of music therapy (30/35); 83.6% of those who attended healing ceremonies (5/6); and 83.3% of spiritual healing practitioners (10/12). The second reason for using CAM was for chronic health problems, which was the most frequent answer among herbal medicine users (40.9%, 27/66) (Figure 3).

### 3.4. Perception of Benefit of Using CAM

**Figure 4:**
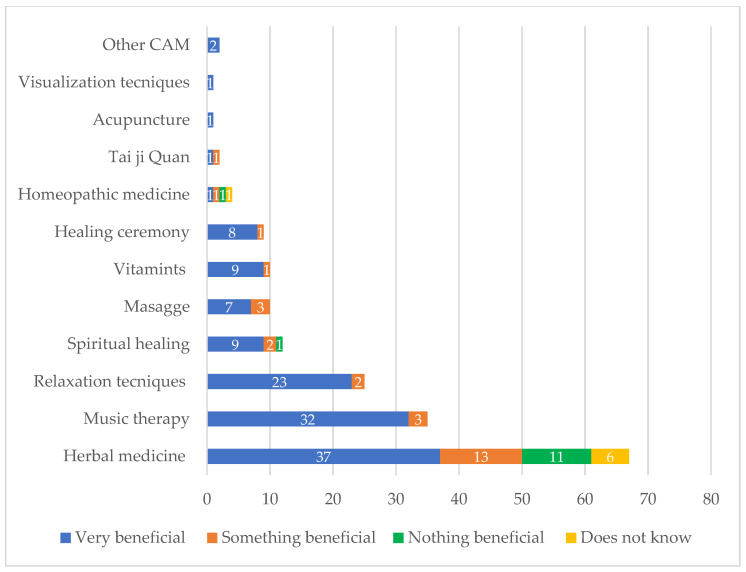
Perception of benefit of using complementary and alternative medicine (CAM) in patients.

Regarding the question the benefits attributed to the use of CAM, of the 184 responses, the majority indicated its use was very beneficial (73.3%; 135/184). This was specifically reported by users of relaxation techniques (92.0%; 23/25) and music therapy (90.6%; 29/32), those who take vitamins (90.0%; 9/10), and those who engage in spiritual healing 75.0% (9/12). In contrast, fewer patients consuming herbal plants (56.1%; 37/66) indicated their use was very beneficial (Figure 4).

### 3.5. Adverse Effects and Starting Time of CAM Use

Overall, 97.0% (128/132) of CAM users stated that their use did not cause side effects, while the remaining 3% (4/132) reported secondary effects on gastrointestinal tract (2/4) and nervous system (2/4). Regarding the start time of their utilization of CAM, 54.5% (72/132) of patients noted prior use, and 45.5% (60/132) began use after starting treatment with peritoneal dialysis.

### 3.6. Recommending the Use of CAM

The main sources of recommendation for use of CAM were family members (40.1%; 53/132), followed by friends (22.7%; 30/132), allopathic doctors (8.3%; 11/132), other health professionals (6% 8/132), and marketing (2.2%; 3/132).

### 3.7. Inform the Use of CAM to Medical Doctor

Only 31.8% (42/132) of CAM users reported to their doctors about use of CAM. The remaining 68.2% (90/132) of the users did not report it for the following reasons: (a) the doctor did not ask (72.2%, 65/90), (b) the patients did not consider it necessary (20.0%; 18/90), (c) the patients did not provide information for fear of disapproval (6.7%; 6/90), and (d) the patients did not have medical assistance at the time they used CAM (1.1%; 1/90).

### 3.8. Clinical Characteristics and Biochemical Parameters of Patients

The duration of PD therapy in the participating patients ranged from 1 to 168 months, with a mean of 27.4 months (±27.6), and no statistically significant difference was observed between the months of PD and CAM use or not (*p* = 0.412). The average volume of residual uresis in our study population ranged from 0 to 3000 mL, with a mean of 649 mL (±564), and no statistically significant difference was observed between the volume of residual uresis and the CAM users or non-users of CAM (*p* = 0.447).

Table 4 and Table 5 display the clinical characteristics and biochemical parameters of CAM users and non-users, respectively. We did not find significant differences in clinical and biochemical characteristics between the two groups; only the diastolic pressure was significant significantly higher in CAM users (Table 5). On the other hand, most of the patients were overweight (44.2%; 106/240) or obese (26.3%; 63/240). According to levels of albumin and BMI, 72.9% (175/240) had adequate nutritional status. Further, 60% of patients (144/240) had a Karnosfsky index higher than 80 points, which suggests that they were able to independently carry out daily activities. The main etiology of CKD reported was diabetic nephropathy (62.5%; 150/240), and the patients had between two and seven comorbidities.

## 4. Discussion

The use of CAM has increased in recent decades, mainly for the prevention and management of chronic diseases and the well-being needs of the older population.

In recent years, the WHO has implemented a strategy for integration, validation, and safety to harness the potential contribution of CAM to health, wellness, and people-centered health care [27]. In Mexico, native peoples have a wide tradition of CAM use. However, studies on CAM use in chronic diseases are scarce. Our study explored the prevalence of CAM use in patients with CKD treated with PD, the types and reasons for its usage, the perception of its benefit, and its adverse effects.

Fifty-five percent of our study population was identified to be using CAM therapy; this finding was similar to the reports of a study in a German population, where 57% of dialysis patients reported to be regular CAM consumers [28]. Studies in a Turkish (72.5%) [29] population showed high frequencies of CAM use; on the other hand, studies in American (18.0%) [24] patients reported lower frequencies. The use of CAM can vary by diverse demographic factors such as age, sex, educational status, socioeconomic status, and occupational status [30]; however, in our study, none of the demographic factors analyzed had a significant influence on CAM. Women are more likely to use CAM than men [31], but we did not find a sex effect on CAM use for CKD in our study. However, studies in patients with kidney disease showed that both men and women are likely to use CAM; in studies in Egyptian [32] and Indian [33] patients, men were more likely to use CAM, whereas studies in Saudi patients showed that women are more likely [34]. Regarding the activity of our patients that identified as CAM users, 24% were housewives, followed by retirees (17.5%), patients with only primary education, and patients with medium-low (21.3%) and medium (22.1%) socioeconomic levels.

Medicinal plants are part of the therapeutic resources of traditional pre-Hispanic medicine in Mexico, and these are culturally and historically popular [35], so it is not unusual for herbal medicine (50.0%) to be the most common type of CAM used by patients. Similar findings were reported in American (67.8%) [24] and Turkish (76.9%) [29] patients; however, it differs from that reported in German patients, whose most common type of CAM was mineral supplements [28]. Occasionally, factors such as culture, history, idiosyncrasies, and beliefs influence the use of different CAM types [36]. On the other hand, 34.8% (46/132) of our patients employed more than one type of CAM-even up to six CAM; in German patients, 27.0% employed more than one CAM, and patients reported using up to five CAM.

Herbal medicines can include a variety of potentially hepatotoxic compounds: (a) natural products such as volatile compounds, glycosides, terpenoids, alkaloids, anthraquinones, phenolics compounds, and other toxins; (b) contaminants or adulterants such as metals, mycotoxins, and pesticides; and herbicidal residues, and their mechanism to induce hepatotoxicity remains mostly imprecise in many cases [37]. In addition, herbal medicines can carry a variety of nephrotoxic compounds such as organic acids, alkaloids, terpenes, lactones, saponins, indeed minerals, and toxic proteins [38]. The use of herbal medicines by CKD patients is especially detrimental because of hepatotoxicity and nephrotoxicity, hemodynamic changes, electrolyte abnormalities, and effects on blood pressure, blood glucose, and coagulation parameters [29,30]. With the increasing of use of herbal medicines, there is a need to monitor and study their safety, especially in patients with CKD. In fact, the WHO recommends including the herbal medicine pharmacovigilance systems [39]. Chamomile (25.8%, 25/97) and moringa leaves (16.0%, 15/97) were the most common herbs used by patients, and various studies have shown the beneficial effects and low side effects of both plants [40,41].

Improving well-being (71.7%; 132/184) was the most frequent reason for using CAM in this study, unlike American patients, who use CAM to improve their energy and concentration [24], and German and Turkish patients, who use it to strengthen their immune system [28,29]. The majority of CAM (73.3%; 135/184) referred to by patients was considered as beneficial, which is similar to the report by Duncan et al. in American patients (77.8%) [24] but less so in Turkish patients (95.5%) [29]. With respect to side effects, 95.4% (126/132) of CAM users did not present adverse effects; however, in a Turkish study, a smaller number of patients (77.3%) did not experience side effects of CAM, probably due to the fact that Turkish patients used more herbal plants, or the plants employed by the patients had undesirable effects [29].

In our study, similar to that reported by Uzdil and Kılıç, the majority of people who recommended CAM were family members and friends. In addition, this investigation reported that 81.6% of patients recommended CAM to another person [29].

A low number of patients (31.8%; 42/132) informed their physicians about CAM consumption compared to German (59%) [28] and American (36.8%) [24] patients. Physicians not asking patients about the use of CAM was the main reason for patients not informing physicians, which reflects the poor interest of medical doctors in the use of CAM. This interest needs to be improved because, as shown before, herbal plants are the most common type of CAM referred by patients, and CKD patients use many drugs for different complications at the same time, and interactions between drugs and herbs may mimic, decrease, or increase the action of prescribed drugs [30,42]. Improving patient–physician communication is essential for positive health outcomes. The lack of adequate discussion about CAM use raises the risk of adverse effects, including interactions with conventional treatments, which could be related to social perceptions [22,43].

All patients included in the study had clinical and biochemical characteristics; previous studies in the literature did not consider these parameters; therefore, we considered these as contributions. Most participants were overweight or obese, and increasing evidence suggests that obesity is a risk factor for diabetes and CKD, and high BMI has been reported to be related to diabetic nephropathy [44]. These data are consistent with the findings of our study, for which the main etiology of CKD was diabetic nephropathy (62.5%; 150/240).

According to levels of serum albumin and BMI, 72.9% (175/240) of patients showed good nutritional status; in addition, other biochemical parameters were analyzed, such as hemoglobin, urea, creatinine, glucose, uric acid, calcium, phosphorus, and potassium, and we did not observe significant differences between users and non-users of CAM. These results indicate that CAM use does not have a negative effect on the health of patients with CKD. In addition, no significant differences were observed in either group with respect to edema grade or systolic pressure, suggesting that the use of CAM is not associated with changes in fluid status in patients with CKD on PD. In contrast, the diastolic pressure was significantly higher in CAM users; however, we believe that this is not clinically relevant.

The patients in our study had between two and seven comorbidities such as acute myocardial infarction, heart failure, peripheral vascular disease, dementia, chronic lung disease, connective tissue diseases, peptic ulcer disease, liver diseases, HIV, and diabetes mellitus, and according to the comorbidity scale, no significant differences were observed between users (score = 3) and non-users (score = 3.1) of CAM. Contrary to other studies, CAM users have a greater number of diseases [28,45].

This investigation has limitations: as a cross-sectional design, the conclusions drawn from the study cannot suggest causation and only included patients from the unique dialysis clinic of one hospital; therefore, our results may not reveal CAM use in other provinces considering the wide difference in culture, beliefs, and idiosyncrasies of Mexico. Despite these limitations, our results provide an important new understanding, and to the best of our knowledge, this is the first study on the use of CAM in CKD patients in Mexico.

## 5. Conclusions

The use of CAM is popular among renal patients on PD (55%), with the main type of herbal medicine being chamomile, followed by relaxation as part of the practice of mind and body techniques. The main reason for the use of CAM in our patients was to improve their state of well-being, and only 3% of users reported side effects. Just as 31.8% of the users of CAM informed their doctor, we need continued research and education to identify and break down barriers to the communication of CAM-use topics between patient and doctor, as this is mandatory.

## Figures and Tables

**Table 1:** Sociodemographic characteristics of patients.

Variables	CAM Users	CAM Non-Users	Total	*p*-Value
	*n* (%)	*n* (%)	*n* (%)	
**Sex**				
Female	65 (27.1)	64 (26.7)	129 (53.8)	0.122
Male	67 (27.9)	44 (18.3)	111 (46.3)	
**Occupation**				
Employed	4 (1.7)	6 (2.5)	10 (4.2)	0.210
Housewife	49 (20.4)	39 (16.3)	88 (36.7)	
Professional	0 (0.0)	2 (0.8)	2 (0.8)	
Retired	42 (17.5)	27 (11.3)	69 (28.8)	
Unemployed	32 (13.3)	33 (13.8)	65 (27.1)	
Other	5 (2.1)	1 (0.4)	6 (2.5)	
**Education level**				0.174
No formal education	6 (2.5)	14 (5.8)	20 (8.3)	
Able to read and write	5 (2.1)	4 (1.7)	9 (3.8)	
Primary education	59 (24.6)	50 (20.8)	109 (45.4)	
Secondary education	28 (11.7)	17 (7.1)	45 (18.8)	
High school	21 (8.8)	13 (5.4)	34 (14.2)	
Bachelor’s degree	13 (5.4)	9 (3.8)	22 (9.2)	
Higher	0 (0.0)	0 (0.0)	0 (0.0)	
**Socioeconomical level**				0.857
Low	4 (1.7)	3 (1.3)	7 (2.9)	
Low high	10 (4.2)	10 (4.2)	20 (8.3)	
Medium low	51 (21.3)	47 (19.6)	98 (40.8)	
Medium	53 (22.1)	35 (14.6)	88 (36.7)	
Medium high	12 (5.0)	12 (5.0)	24 (10.0)	
High	2 (0.8)	1 (0.4)	3 (1.3)	

Statistically significant. *p*-value was calculated using chi-square test. CAM, complementary and alternative medicine.

**Table 2:** Distribution of types of CAM used by patients.

Type CAM	*n* = 132 (%)
**Herbal medicine**	66 (50.0)
**Mind-body practices**	
Music therapy	32 (24.2)
Relaxation techniques	25 (18.9)
Meditation	9 (6.8)
Stretching techniques	3 (2.3)
Visualization techniques	1 (0.8)
**Spiritual healing**	12 (9.1)
Massage/articular therapy	10 (7.6)
Vitamins and/or minerals	10 (7.6)
Healing ceremony	6 (4.5)
Homeopathic medicine	4 (3.0)
Tai ji Quan	2 (1.5)
Acupuncture	1 (0.8)
**Magnetic therapy**	
Magnets	1 (0.8)
Chakras	1 (0.8)
**Ozone therapy**	1 (0.8)

CAM, complementary and alternative medicine.

**Table 3:** Herbal medicine employed by patients.

Type of Herbal Medicine	*n* = 97 (%)
Chamomile (*Matricaria chamomilla*)	25 (25.8)
Moringa leaves (*Moringa oleifera*)	15 (15.5)
Chaya leaves (*Cnidoscolus aconitifolius*)	8 (8.2)
Orange leaves (*Citrus aurantium*)	6 (6.2)
Linden *(Tilia mexicana)*	4 (4.1)
Azhar (*Orange blossom*)	2 (2.1)
Noni fruit (*Morinda citrifolia*)	2 (2.1)
Neem (*Azadirachta indica*)	2 (2.1)
Lemon *(Citrus limon)*	2 (2.1)
Riñonina (*Ipomoea pes-caprae*)	2 (2.1)
Cinnamon (*Cinnamomum verum*)	2 (2.1)
Oregano (*Plectranthus amboinicus*)	2 (2.1)
Rue (*Ruta chalepensis*)	2 (2.1)
Blue agave (*Agave tequilana*)	1 (1.0)
Belladonna (*Kalanchoe integra*)	1 (1.0)
Bougainvillea (*Bougainvillea glabra*)	1 (1.0)
Espada de cristo *(Dracaena trifasciata)*	1 (1.0)
Peppermint (*Mentha x piperita*)	1 (1.0)
Hibiscus flower (*Hibiscus rosa-sinensis*)	1 (1.0)
Laurel (*Ficus benjamina*)	1 (1.0)
Cow tongue (*Dracaena trifasciata*)	1 (1.0)
Blue berry (*Vaccinium corymbosum*)	1 (1.0)
Cucumber cat (*Parmenteria aculata*)	1 (1.0)
Beetroot (*Beta vulgaris*)	1 (1.0)
Sabila (*Aloe vera*)	1 (1.0)
Clove (*Syzygium aromaticum*)	1 (1.0)
Unknown tincture	1 (1.0)
Acacia (*Acacia cornigera*)	1 (1.0)
Aloe (*Aloe vera*)	1 (1.0)
Coriander (*Coriandrum sativum*)	1 (1.0)
Coconut (*Cocos nucifera*)	1 (1.0)
Lettuce (*Lactuca sativa* Yucateca)	1 (1.0)
Valerian (*Valeriana officinalis*)	1 (1.0)
Basil (*Ocimum basilicum*)	1 (1.0)
Añil (*Indigofera tinctoria*)	1 (1.0)
Chia (*Salvia hispanica*)	1 (1.0)

**Table 4:** Clinical characteristics of patients.

Variables	CAM Users	CAM Non-Users	Total	*p*-Value
	*n* (%)	*n* (%)	*n* (%)	
**Body mass index**				0.816
Underweight	2 (0.8)	1 (0.4)	3 (1.3)	
Normal	37 (15.4)	31 (12.9)	68 (28.3)	
Overweight	54 (22.5)	52 (21.7)	106 (44.2)	
Obesity type 1	28 (11.7)	18 (7.5)	46 (19.2)	
Obesity type 2	8 (3.3)	4 (1.7)	12 (5.0)	
Obesity type 3	3 (1.3)	2 (0.8)	5 (2.1)	
**Edema grade**				0.370
Without edema	45 (18.8)	45 (18.8)	90 (37.5)	
Grade 1	38 (15.8)	35 (14.6)	73 (30.4)	
Grade 2	33 (13.8)	17 (7.1)	50 (20.8)	
Grade 3	13 (5.4)	10 (4.2)	23 (9.6)	
Grade 4	3 (1.3)	1 (0.4)	4 (1.7)	
**Nutritional status**				0.422
With malnutrition	33 (13.8)	32 (13.3)	65 (27.1)	
Without malnutrition	99 (41.3)	76 (31.7)	175 (72.9)	
**Karnosfsky index**				0.977
100	15 (6.3)	9 (3.8)	24 (10.0)	
90	37 (15.4)	31 (12.9)	68 (28.3)	
80	27 (11.3)	25 (10.4)	52 (21.7)	
70	25 (10.4)	20 (8.3)	45 (18.8)	
60	15 (6.3)	13 (5.4)	28 (11.7)	
50	13 (5.4)	10 (4.2)	23 (9.6)	
**Peritoneal dialysis type**				0.264
CAPD	74 (30.8)	59 (24.6)	133 (55.4)	
APD	38 (15.8)	39 (16.3)	77 (32.1)	
IPD	20 (8.3)	10 (4.2)	30 (12.5)	
**Etiology of CKD**				0.334
Diabetic nephropathy	84 (35)	66 (27.5)	150 (62.5)	
Nephroangiosclerosis	14 (5.8)	10 (4.2)	24 (10.0)	
Lithiasis	16 (6.7)	8 (3.3)	24 (10.0)	
Glomerulonephritis	0 (0)	1 (0.4)	1 (0.4)	
Polycystic kidney disease	3 (1.3)	2 (0.8)	5 (2.1)	
Lupus	2 (0.8)	1 (0.4)	3 (1.3)	
No determination	6 (2.5)	14 (5.8)	20 (8.3)	
Other	7 (2.9)	6 (2.5)	13 (5.4)	
**Renal transplantation protocol**				0.410
Yes, living donor	6 (2.5)	2 (0.8)	8 (3.3)	
Yes, deceased donor	27 (11.3)	19 (7.9)	46 (19.2)	
No	94 (39.2)	87 (36.3)	181 (75.4)	

*p*-value was calculated using chi-square test. CAM, complementary and alternative medicine; CKD, chronic kidney disease; CPDA, continuous ambulatory peritoneal dialysis; APD, automated peritoneal dialysis; IPD, intermittent peritoneal dialysis.

**Table 5:** Biochemical parameters and clinical characteristics of patients.

Variables	CAM Users				CAM Non-Users				*p*-Value
	Mean	Minimum	Maximum	±SD	Mean	Minimum	Maximum	±SD	
Hemoglobin (g/dL)	10.2	7	15	1.6	10	6	16.9	2	0.841
Urea (mg/dL)	130.1	42	302	46.9	128	13	336	55.4	0.814
Creatinine (mg/dL)	10.2	2.8	22.7	4	10.4	1.2	26.8	4.49	0.996
Glucose (mg/dL)	125.5	9.4	398	65	122	47	328	48.8	0.615
Uric acid (mg/dL)	6.5	2.4	12.7	1.5	6.9	2.8	11.5	1.63	0.094
Albumin (gr/dL)	3.3	1.9	4.8	0.5	3.2	1.6	4.9	0.59	0.558
Calcium (mg/dL)	8.8	6.9	11.4	0.7	8.9	7	11.6	0.76	0.715
Phosphorus (mg/dL)	5.4	2	11.4	1.5	5.3	2.5	11.5	1.9	0.729
Potassium (mg/dL)	4.3	2.8	7.2	0.7	4.3	2.9	6.8	0.7	0.227
Systolic pressure (mmHg)	134	100	220	19.8	133	90	200	17.9	0.525
Diastolic pressure (mmHg)	82	50	130	11.1	78.4	60	100	9.7	0.005 *
Charlson Comorbidity Index	3.0	2.0	7	0.9	3.1	2	6	0.98	0.519

* Statistically significant. *p*-value was calculated using chi-square test. CAM, complementary and alternative medicine.

## Data Availability

The datasets generated and analyzed in the current study are not publicly available because they are the property of the Instituto Mexicano del Seguro Social. Institutional and federal bodies restrict unlimited access to personal data, but they are available from the corresponding authors upon reasonable request with prior authorization from the institution.

## Supplementary Materials

The following supporting information can be downloaded at: https://www.mdpi.com/article/10.3390/healthcare11050722/s1, File S1. The International Complementary and Alternative Medicine Questionnaire.
