# Supplementary material for: The Use of Complementary and Alternative Medicine among Peritoneal Dialysis Patients at a Second-Level Hospital in Yucatán Mexico
Source: Healthcare (Basel). 2023 Mar 1;11(5):722. doi: 10.3390/healthcare11050722 (PMC10001407; doi:10.3390/healthcare11050722)
Supplement: Supplementary file 1 [file healthcare-11-00722-s001.zip › healthcare-2235960-supplementary.pdf]

ANEXO 1

USANZA DE MEDICINA ALTERNATIVA Y COMPLEMENTARIA POR PACIENTES CON ENFERMEDAD RENAL CRÓNICA TERMINAL EN TRATAMIENTO CON DIÁLISIS PERITONEAL ATENDIDOS EN EL HOSPITAL GENERAL REGIONAL No.1 DEL INSTITUTO MEXICANO DEL SEGURO SOCIAL, DELEGACIÓN YUCATÁN

ENCUESTA DE PACIENTES DEL SERVICIO DE DIÀLISIS PERITONEAL AMBULATORIA DEL HGR No. 1 IMSS

Entrevistado Clave: ________________________Entrevistador: ________________________ Fecha de la entrevista: ___________________

**A. CONSULTAS A PROFESIONALES O EXPERTOS**. LOS PROBLEMAS DE SALUD PUEDEN SER TRATADOS POR DIFERENTES PROFESIONALES. QUISIERA HACERLE ALGUNAS PREGUNTAS SOBRE ESTO:																
PROFESIONAL O EXPERTO EN CAM				¿Cuál fue el motivo principal por el que Ud. consultó a un_________ por última vez?							¿Cuán beneficioso le resultó ver a un _________? ¿Diría que…..?					
En los últimos 12 meses ¿ha consultado a un______?	Si	No	¿No. de veces en los últimos 3 meses?	Por un problema de salud agudo, es decir, uno que duró menos de un mes	Un problema de salud crónico, es decir, uno que duró más de un mes, ya sea para tratar el problema en sí, o sus síntomas	Para mejorar su bienestar	Otra, por favor especificar	No corresponde	No sabe	No contesta	Muy beneficioso	Algo beneficioso	Para nada beneficioso	No corresponde	No sabe	No contesta
1. Médico	☐	☐	______	☐	☐	☐		☐	☐	☐	☐	☐	☐	☐	☐	☐
2. Quiropráctico	☐	☐	______	☐	☐	☐		☐	☐	☐	☐	☐	☐	☐	☐	☐
3. Homeópata	☐	☐	______	☐	☐	☐		☐	☐	☐	☐	☐	☐	☐	☐	☐
4. Acupunturista	☐	☐	______	☐	☐	☐		☐	☐	☐	☐	☐	☐	☐	☐	☐
5. Fitoterapeuta o yerbatero	☐	☐	______	☐	☐	☐		☐	☐	☐	☐	☐	☐	☐	☐	☐
6. Sanador espiritual	☐	☐	______	☐	☐	☐		☐	☐	☐	☐	☐	☐	☐	☐	☐
7. Otro tipo de experto. ¿Qué tipo?	☐	☐	______	☐	☐	☐		☐	☐	☐	☐	☐	☐	☐	☐	☐

B. TRATAMIENTOS REALIZADOS POR PROFESIONALES O EXPERTOS. QUISIERA PREGUNTAR SOBRE TRATAMIENTOS QUE HAYA RECIBIDO DE PARTE DEL PROFESIONAL O EXPERTO.

En los últimos 12 meses ¿ha recibido tratamiento de?	Si	No	¿No. de veces en los últimos 3 meses?	¿Cuál fue el motivo principal por el que Ud. recibió el tratamiento de_________ por última vez?						
				Por un problema de salud agudo, es decir, uno que duró menos de un mes	Un problema de salud crónico, es decir, uno que duró más de un mes, ya sea para tratar el problema en sí, o sus síntomas	Para mejorar su bienestar	Otra, por favor especificar	No corresponde	No sabe	No contesta
1. Manipulación articular y/o masaje			______							
2. Homeopatía			______							
3. Acupuntura			______							
4. Hierbas, tés, infusiones o tinturas			______							
5. Sanacion espiritual			______							
Otro tipo de tratamiento. ¿Qué tipo?			______							
7. Participar de una ceremonia de sanación tradicional			______							
8. Rezar por su salud			______							
9. Utilizó alguna otra práctica personal. ¿Qué tipo?			______							

En los últimos 12 meses ¿ha recibido tratamiento de?	¿Cuan beneficioso le resultó el tratamiento de _________? ¿Diría que…..?					
	Muy beneficioso	Algo beneficioso	Para nada beneficioso	No corresponde	No sabe	No contesta
1. Manipulación articular y/o masaje						
2. Homeopatía						
3. Acupuntura						
4. Hierbas, tés, infusiones o tinturas						
5. Sanacion espiritual						
Otro tipo de tratamiento. ¿Qué tipo?						
7. Participar de una ceremonia de sanación tradicional						
8. Rezar por su salud						
9. Utilizó alguna otra práctica personal. ¿Qué tipo?						

C. USO DE MEDICAMENTOS A BASE DE HIERBAS Y SUPLEMENTOS DIETÉTICOS. ADEMÁS DE LOS MEDICAMENTOS QUE LE PRESCRIBEN LOS MÉDICOS, ALGUNAS PERSONAS UTILIZAN GRAN VARIEDAD DE OTROS PRODUCTOS PARA LA SALUD, COMO POR EJEMPLO MEDICAMENTOS A BASE DE HIERBAS Y SUPLEMENTOS DIETÉTICOS QUE PUEDEN SER COMPRIMIDOS, CÁPSULAS, O LÍQUIDOS (TÉS, TINTURAS, JARABES, INFUSIONES, ETC.)

En los últimos 12 meses ¿ha recibido ingerido medicamentos a base de hierbas o suplementos?	Si	No	¿No. de veces en los últimos 3 meses?	¿Cuál fue el motivo principal por el que Ud. recibió usó medicamentos_________ por última vez?							¿Cuan beneficioso le resultó el uso de medicamentos a base de hierbas o suplementos _________? ¿Diría que.....?					
				Por un problema de salud agudo, es decir, uno que duró menos de un mes	Un problema de salud crónico, es decir, uno que duró más de un mes, ya sea para tratar el problema en sí, o sus síntomas	Para mejorar su bienestar	Otra, por favor especificar	No corresponde	No sabe	No contesta	Muy beneficioso	Algo beneficioso	Para nada beneficioso	No corresponde	No sabe	No contesta
Hierba 1 ___________			______													
Hierba 2 ___________			______													
Hierba 3 ___________			______													
Vitamina/ mineral 1			______													
Vitamina/ mineral 2			______													
Vitamina/ mineral 3			______													
Remedio homeopático 1			______													
Remedio homeopático 2			______													
Remedio homeopático 3			______													

Otro Especificar:			______													

D. PRÁCTICAS PERSONALES QUE PROMUEVAN EL BIENESTAR. ADEMÁS DE LOS MEDICAMENTOS QUE PRESCRIBEN LOS MÉDICOS ALGUNAS PERSONAS SUELEN UTILIZAR DIFERENTES TIPOS DE PRÁCTICAS PARA MEJORAR LA SALUD. HA REALIZADO ALGUNA DE ÉSTAS EN LOS ÚLTIMOS 12 MESES																
En los últimos 12 meses ¿ha realizado prácticas personales como?	Si	No	No. de veces en los últimos 3 meses?	Cuál fue el motivo principal por el que Ud. realizó la práctica_________ por última vez?							Cuan beneficioso le resultó la realización de la práctica _________? ¿Diría que…..?					
				Por un problema de salud agudo, es decir, uno que duró menos de un mes	Un problema de salud crónico, es decir, uno que duró más de un mes, ya sea para tratar el problema en sí, o sus síntomas	Para mejorar su bienestar	Otra, por favor especificar	No corresponde	No sabe	No contesta	Muy beneficioso	Algo beneficioso	Para nada beneficioso	No corresponde	No sabe	No contesta
1.Mediatación			______				______									
2. Yoga			______				______									
3. Qi Gong (Chi Kung)			______				______									
4. Tai Ji Quan (Tai Chi Chuan)			______				______									
5. Técnicas de relajación			______				______									
6. Visualización			______				______									
Otro Especificar: ________			______				______									

**OTRA INFORMACION**<br>Momento de inicio de uso de CAM ( ) Antes de DP ( ) Despúés de DP	Efectos adversos al uso de MAC<br>SI ( ) NO ( )
¿Ha informado a su médico el uso de medicina alternativa y/o complementaria?<br>SI ( ) NO ( )	Tipo de efectos adversos percibidos:<br>( ) Gastrointestinales ( ) Sistema nervioso ( ) Dermatológico ( ) toxicológicos ( ) en vías urinarias

Informa a su médico ( ) Si ( ) No<br><br>Razones por las que no informa a su médico<br><br>( ) No me pregunta ( ) No es necesario ( ) temor a desaprobación ( ) Otro _________	Recomendado por:<br><br>( ) Médico institucional ( ) Médico particular ( ) Otros profesionales de la salud ( ) otros pacientes<br><br>( ) Familiares ( ) Amigos ( ) Publicidad/medios de comunicación ( ) Otros
